# Leveraging radiotherapy to improve immunotherapy outcomes: rationale, progress and research priorities

**DOI:** 10.1002/cti2.70030

**Published:** 2025-04-08

**Authors:** Faith Hartley, Martin Ebert, Alistair M Cook

**Affiliations:** ^1^ Medical School University of Western Australia Perth WA Australia; ^2^ National Centre for Asbestos Related Diseases, Institute for Respiratory Health University of Western Australia Nedlands WA Australia; ^3^ School of Physics Mathematics and Computing University of Western Australia Perth WA Australia; ^4^ Department of Radiation Oncology Sir Charles Gairdner Hospital Nedlands WA Australia; ^5^ School of Biomedical Sciences University of Western Australia Nedlands WA Australia

**Keywords:** immunotherapy, radiotherapy, T cells, translational immunology, tumor immunology

## Abstract

The most successful immunotherapies for solid malignancies to date, immune checkpoint inhibitors, target the essential role of T cells in antitumor immunity. However, T‐cell dysfunction presents a major hindrance to treatment efficacy, warranting research into combined treatment strategies for improving outcomes. The use of radiotherapy for this purpose has garnered much interest. Preclinical study has established that radiotherapy activates various immune mechanisms to improve T‐cell activation, localisation and function within tumors, which improves response to immune checkpoint inhibitors. However, so far, these strategies have not been successfully translated into the clinic. Here, we briefly reflect on the development of immune checkpoint inhibitors and the mechanistic insights revealed by an evolving understanding of T‐cell dysfunction in cancer, before providing an overview of the immunomodulatory effects of radiotherapy in the context of the T‐cell‐mediated antitumor immune response. We discuss the mixed results of clinical trials, comment on various factors that may preclude immuno‐radiotherapy responses in the clinic, and highlight priorities for preclinical and clinical study. Finally, we discuss the role of emerging combinations of radiotherapy and immunotherapy to potentially provide additional treatment options and improve outcomes for patients.

## Graphical abstract

While immune checkpoint inhibitors have shown remarkable success in solid tumours, the induction of T cell dysfunction limits their effectiveness, resulting in treatment resistance and relapse. This has prompted interest in combination strategies, such as taking advantage of the immunomodulatory capabilities of radiotherapy, to improve responses; however, clinical translation remains a challenge. This review explores the immune effects of radiotherapy, mixed results of clinical trials, barriers to translation, and finally emerging combinations of radiotherapy and immunotherapy.

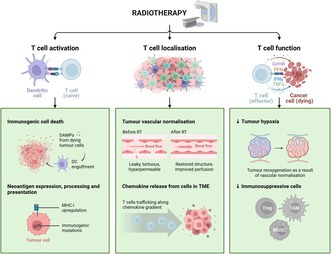

## Introduction

The most successful immunotherapies for solid malignancies to date, immune checkpoint inhibitors (ICI), target the essential role of T cells in antitumor immunity. Recent efforts to characterise the dysfunctional states of T cells in cancer have yielded further insights into the mechanisms and limitations of ICI. For example, many groups have demonstrated the importance of ‘precursor‐exhausted’ or ‘stem‐like’ T cells in the response to anti‐PD1/anti‐PD‐L1 ICI, which undergo a proliferative burst following treatment that drives immune‐mediated destruction of tumor cells.^1^ However, tumors are typically dominated by a ‘terminally exhausted’ CD8^+^ T‐cell population, which arises from these precursor subsets under pressure from the immunosuppressive tumor microenvironment (TME), and is marked by irreversible dysfunction and resistance to ICI.^1^ Accordingly, many patients derive no benefit from, or acquire resistance to, ICI—highlighting a need for combinatorial strategies to improve ICI efficacy.

Radiotherapy (RT) is a potent inducer of DNA damage and cell death but also activates immune mechanisms that can enhance activation, infiltration and function of T cells. Various studies report that RT enriches for precursor‐exhausted CD8^+^ T cells within tumor and enhances T‐cell receptor (TCR) clonality,^2^, ^3^, ^4^ indicating activation of a tumor‐specific immune response that can support ICI activity. However, while some clinical trials assessing immuno‐radiotherapy (IO‐RT) combinations have shown encouraging efficacy, others have failed to demonstrate therapeutic benefit. In part, this is because of opposing immunosuppressive effects also exerted by RT; clinical trials to date have highlighted additional considerations for optimising IO‐RT, including overall tumor burden and lymph node (LN) involvement. As current IO‐RT strategies only benefit a subset of patients, there is a need to identify biomarkers predictive of response. Equally important is the need to investigate combining RT with emerging immunotherapeutic strategies, such as CAR‐T cell therapy and cancer vaccines, to expand the range of viable treatment options for patients.

In this review, we briefly reflect on the development of ICI and the mechanistic insights revealed by an evolving understanding of T‐cell dysfunction in cancer, before providing an overview of the immunostimulatory and immunosuppressive effects of RT in the context of the T‐cell‐mediated antitumor immune response. We then focus on the results of selected IO‐RT clinical trials and discuss factors that improve or preclude responses in the clinic. Finally, we discuss emerging IO‐RT combinations as potential avenues for improving the rate and durability of treatment response.

## Immunotherapy: Exploiting the essential role of T cells in antitumor immunity

The canonical adaptive cancer immunity cycle emphasises the critical function of T cells, particularly cytotoxic CD8^+^ T cells, in orchestrating tumor cell killing.^5^ Significant advancements have been made with immunotherapeutic strategies targeting these T cells, growing out of an in‐depth understanding of how T cells are activated and maintain their function. For example, a core concept in successful T‐cell priming is the requirement of three signals: (1) TCR engagement with its cognate antigen expressed on an MHC molecule, (2) binding of co‐stimulatory molecules and (3) release of cytokines.^5^ ICI augments the second of these signals by blocking co‐inhibitory immune checkpoint molecules (e.g. PD1, CTLA4).^5^ Ongoing efforts to define T‐cell heterogeneity and the evolution of T‐cell phenotype in the TME have yielded further mechanistic insights; in particular, much attention has been given to characterising programs of T‐cell dysfunction such as exhaustion and stress.

**Figure 1:**
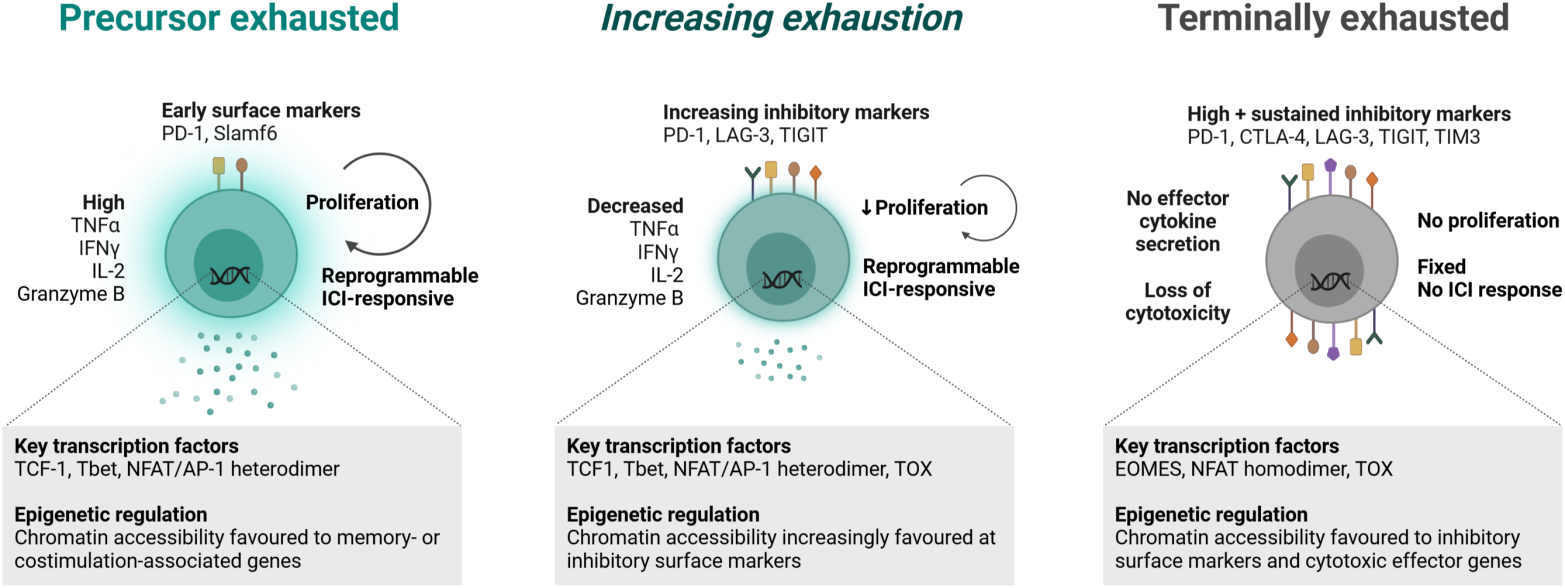
Spectrum of exhausted T cell subsets within the tumor. This figure was created with Biorender.com.

In cancer, T‐cell exhaustion (Figure 1) is defined as the decline in function of tumor‐reactive T cells, characterised by the loss of proliferative capacity, reduced inflammatory cytokine secretion and increased expression of inhibitory receptors. Studies in chronic viral infection and cancer have identified a population of precursors with stem‐like renewal capabilities (owing to expression of the transcription factor TCF1) that replenish a pool of terminally exhausted CD8^+^ T cells. These cells also co‐express PD1 and Slamf6 on their surface, but generally have low expression of additional exhaustion markers such as TIM3.^5^ The origin of these ‘precursor‐exhausted’ T cells (T_PEX_) remains a subject of active investigation. Initial evidence suggested that effector T cells, subject to continual antigen exposure, progressively acquire features of dysfunction until reaching a terminally exhausted phenotype. However, it has also been proposed that naïve CD8^+^ T cells make a fate decision between the effector and exhaustion differentiation trajectories much earlier in the process, within hours of activation.^6^ Importantly, T_PEX_ have been shown to undergo expansion following anti‐PD1/anti‐PD‐L1 ICI, generating a pool of tumor‐reactive cells with revitalised effector capabilities.^5^ However, terminally exhausted CD8^+^ T cells exhibit loss of TCF1 and elevated expression of PD1 and other exhaustion markers such as LAG3, TIGIT and TIM3; these cells are in a transcriptionally and epigenetically ‘fixed’ state, and antitumor functions are not restored by ICI.^5^ Terminal exhaustion is driven by high expression of the transcription factor TOX, which increases chromatin accessibility of regions encoding several of the inhibitory immune receptors.^7^

T cells with a stress response phenotype (T_STR_) comprise a distinct dysfunctional state that is associated with more aggressive cancer phenotypes and resistance to immunotherapy.^8^ The T_STR_ state was first described by Chu and colleagues, who developed a comprehensive T cell atlas from a total of 27 single‐cell transcriptomic datasets across 16 cancer types.^8^ Compared to other T cell subsets described, including other dysfunctional states such as T cell exhaustion, T_STR_ cells followed a distinct differentiation pathway and notably were characterised by increased expression of stress‐associated heat shock genes (*HSPA1A, HSPA1B*). T_STR_ cells were identified within multiple tumour types, existing in lymphoid aggregates within the TME as well as in potential tertiary lymphoid structures. Both before, but particularly after, anti‐PD1/anti‐PD‐L1, significantly more T_STR_ cells was observed in non‐responding tumours when compared to responders. Early evidence also suggests that T_STR_ cells tend to colocalise with areas of hypoxia in tumours.^8^

**Figure 2:**
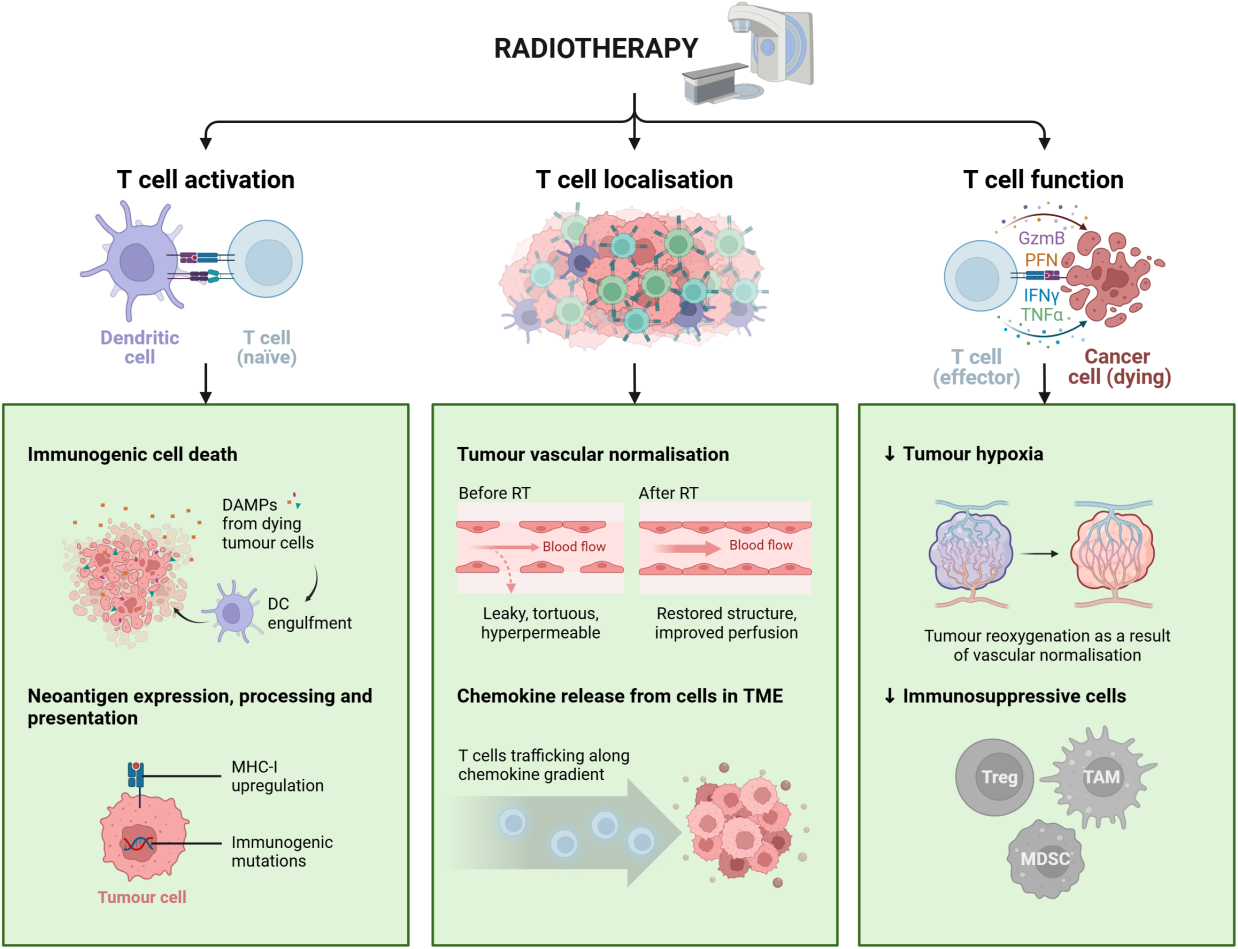
Summary of immunostimulatory effects of radiotherapy. This figure was created with Biorender.com.

The presence of highly dysfunctional T‐cell states within tumors is a major barrier to ICI. The degree of T‐cell dysfunction within tumor is positively associated with tumor burden; therefore, the use of cytoreductive cancer therapies can benefit ICI response. At the same time, given that ICI is largely dependent on newly infiltrating T cells into tumors, it follows that the use of adjunct therapies should be leveraged. Specifically, those with potential to increase expansion of tumor‐reactive T‐cell clones; generate *de novo* activation of T cells; improve T cell trafficking and localisation; or prime the TME to be conducive to T‐cell activity, could help unleash the full potential of ICI. In these respects, it has become increasingly evident that RT can be harnessed not only for its cytoreductive capacities but also to induce immunostimulatory effects to improve T‐cell‐mediated antitumor response and improve response rates to immunotherapies.^9^ Many of these effects have been established through preclinical study, summarised in Figure 2 and elaborated upon below.

## Radiotherapy: Enhancing T‐cell activation, infiltration and function

The ability of RT to activate antitumor immune mechanisms is most impressively displayed in rare, off‐target ‘abscopal’ effects where local irradiation of a tumor leads to regression of distant, unirradiated metastases,^10^ thought to be mediated by CD8^+^ T cells.^11^ The below sections highlight key immunostimulatory mechanisms of RT and the resultant impact on T‐cell‐mediated antitumor immune response.

### Increasing T‐cell activation

Multiple studies point to the potential of RT to increase the activation of tumor‐specific T cells. In a poorly immunogenic model of pancreatic cancer, 5 Gy plus dual ICI (anti‐PD1 and anti‐CTLA4) led to tumor growth delays not seen with either treatment alone; this was accompanied by augmented T‐cell priming as identified by IFN‐gamma (IFNγ) ELISpot in both subcutaneous and orthotopic tumor models.^12^ Furthermore, in human renal cell carcinoma samples, 4 weeks after stereotactic body radiation therapy (SBRT) with a single fraction of 15 Gy, differential gene expression analyses revealed increased levels of effector molecules (IFNG, TNFSF10, PRF1), maturation indicators (CD69, PDCD1) and cell cycle markers (MKI67) in irradiated samples compared with control samples.^13^

Radiotherapy‐induced T‐cell activation can follow the induction of immunogenic cell death (ICD), a form of regulated cell death that results in increased antigen availability.^14^ Key damage‐associated molecular patterns (DAMPs) are associated with ICD. Calreticulin translocation from the endoplasmic reticulum to the cell surface serves to promote dendritic cell (DC) engulfment of dying tumor cells via interaction with its cognate receptor CD91, thus increasing antigen uptake and presentation. Adenosine triphosphate (ATP), actively secreted from dying cells, binds to purinergic receptors (P2RY2 and P2XR7) on DCs, recruiting them to the site of the tumor. Passive HMGB1 release following tumor cell death promotes an inflammatory response and DC maturation, through binding to TLR4 on DCs or tissue macrophages.^15^, ^16^ Furthermore, the presence of cytosolic DNA following RT is sensed by cGAS, which leads to downstream activation of STING and subsequent production of type 1 interferon (IFN‐I); this contributes to DC activation and maturation.^17^, ^18^, ^19^ DCs are crucial for T‐cell activation and T‐cell‐mediated tumor eradication, and in line with this, DC activation has been found to contribute to the therapeutic effect of 10 Gy RT to B16 murine melanoma.^20^, ^21^ Preclinical work provides mixed evidence of dose effects on RT‐induced ICD. In TSA mammary cancer cell lines irradiated with 2 Gy, 5 Gy, 10 Gy or 20 Gy, ICD was found to increase with RT dose.^22^ Following 10 Gy radiation, calreticulin surface expression and HMGB1 and ATP secretion were induced in human breast, lung and prostate cell lines.^23^ However, there is some evidence that higher doses (12–18 Gy) may be detrimental to cGAS/STING activation because of induction of *Trex1*, which degrades cytosolic DNA.^24^ Fractionated schedules may therefore be favorable; 8 Gy × 3 was found to amplify IFN‐beta (IFNβ) production in TSA cells, mediated by cGAS/STING,^25^ while 2 Gy × 5 released ICD‐related DAMPs in glioblastoma cell lines.^16^

Enhancing neoantigen expression, processing and presentation represents an additional means of increasing T‐cell activation. Reits *et al*.^26^ showed that RT increases expression of MHC‐I in a dose‐dependent manner, mediated by RT‐induced degradation of damaged protein, and in later stages, mTOR pathway activation, which leads to increased protein translation and peptide generation.^26^ Numerous other studies have also shown RT‐induced tumor MHC‐I upregulation in different cancers.^21^, ^27^, ^28^ In addition, RT can unmask immunogenic mutations that lead to expression of novel peptides presented by MHC‐I.^26^ RT with 8 Gy × 3 also increased expression of immunogenic mutations in 32 of 34 non‐small‐cell lung cancer (NSCLC) patients.^29^, ^30^

### Enhancing T‐cell localisation

Immunotherapy success largely relies on new T cells infiltrating the tumor, as those persisting within tumors are often already in a state of fixed dysfunction that do not respond to treatment. RT can enhance T‐cell trafficking and recruitment to the tumor.^31^, ^32^ RT‐induced recruitment of stem‐like, TCF1^+^Slamf6^+^ CD8^+^ T cells was essential for response to anti‐PD‐L1 in a poorly immunogenic, T cell excluded model of orthotopic head and neck squamous cell carcinoma (HNSCC).^3^ Mechanistically, RT can induce changes in tumor vasculature and stimulate the release of chemotactic factors to enhance T cell infiltration.

Tumor vasculature is typically leaky and tortuous, leading to stagnant blood flow and poor perfusion that promotes an aggressive, treatment‐resistant tumor phenotype. However, RT has been shown to improve tumor vasculature through functional remodelling.^33^, ^34^ Improved or ‘normalised’ vasculature is associated with massive T‐cell infiltration into tumors,^35^, ^36^ improved delivery of systemic treatments,^37^ reduced tumor hypoxia^32^ and appears to be dose‐dependent. Ablative single or hypofractionated doses have been shown to induce severe vascular damage resulting in reduced perfusion or elevated hypoxia,^38^, ^39^, ^40^ although certain lower dose hypofractionated schedules (4 Gy × 6, 8 Gy × 3) could improve pericyte coverage and reduce hypoxia in a prostate cancer model (PC3).^41^ Numerous studies support the use of 2 Gy daily fractions (2 Gy × 5/week for up to 2 weeks) for remodelling blood vessels.^34^, ^41^, ^42^ A dose titration study in a mesothelioma model (AB1‐HA) found that treatment with 2 Gy × 5 led to the greatest improvements in functional vasculature and CD3^+^ T‐cell numbers in tumors, compared to a lesser number of 2 Gy fractions or a hypofractionated schedule (6 Gy × 2).^34^ The effect on increasing perfusion was transient, observed at 1 and 4 days post RT but not 8 days after, with the degree of CD8^+^ T cell infiltration also reaching a plateau at 4 days.^34^ Importantly, these effects appeared to influence the ideal timing of treatment with 2 Gy × 5 and ICI (anti‐PD1 + anti‐CTLA‐4), as concurrent scheduling led to consistent complete responses (> 90%) with immunological memory, but delaying ICI to 1 week after completion of RT markedly abrogated response rate (14%).^34^

Radiotherapy can upregulate expression and release of chemokines from a range of cells within the TME that are important for effector T‐cell recruitment to tumors. For example, 12 Gy × 2 increased CXCR6^+^CD8^+^ T‐cell infiltration into 4T1 tumors by inducing cancer cell secretion of the pro‐inflammatory chemokine CXCL6.^43^ Low‐dose RT (1 Gy × 3) supported intratumoral infiltration of CD8^+^ T_PEX_ via the CXCL10/CXCR3 axis in orthotopic hepatocellular carcinoma, improving response to anti‐PD‐L1 and anti‐VEGFA.^44^ Furthermore, 20 Gy to mouse sarcoma tumors led to upregulation of CCL2 and CCL5 concurrent with a dramatic increase in CD4^+^ and CD8^+^ T cells in the TME.^45^

### Promoting a favorable tumor microenvironment

The state of the TME is a key mediator in T‐cell function, and RT can alter various aspects of the TME, including the level of hypoxia and the presence of other immune cell subsets, to create an environment conducive to antitumor immunity.

Hypoxia in the TME is linked to resistance to T‐cell infiltration, the presence of terminally exhausted T cells, T‐cell stress and induction of immunosuppressive cell subsets.^8^, ^46^, ^47^ Promoting tumor reoxygenation is therefore a potential strategy to sensitise tumors to immunotherapy.^34^, ^47^ RT has been shown to transiently decrease the level of tumor hypoxia and improve treatment efficacy, closely related to RT‐induced normalisation of the heterogeneous and unstable vasculature in tumors.^37^, ^41^, ^42^ This was observed in AB1‐HA mesothelioma tumors following treatment with 2 Gy × 5; the hypoxic (pimonidazole^+^) area within tumors was significantly reduced for up to 4 days post RT, in line with vascular changes described above.^34^ Furthermore, in an orthotopic model of prostate cancer, 2 Gy × 10 led to progressive decreases in hypoxia levels throughout treatment, and was dramatically reduced at 14 days.^42^ In human squamous cell carcinoma xenografts, 10 Gy led to a decreased hypoxic fraction of tumor, accompanied by increased vessel perfusion. However, hypoxia increased 11 days after treatment, highlighting the importance of the timing of RT schedules and differences between cancer types.^48^

Immunosuppressive cell subsets, including myeloid‐derived suppressor cells (MDSCs) and tumor‐associated macrophages (TAMs), also exacerbate T‐cell dysfunction. Their accumulation within tumors is linked to poor clinical outcomes^49^, ^50^, ^51^; however, in some cases, RT may reduce the numbers or effect of immunosuppressive cells within the TME. TAMs display considerable functional heterogeneity owing to their high plasticity upon immunological stimulation, with both antitumor (pro‐inflammatory, ‘M1‐like’), but mostly pro‐tumor (tissue repair, ‘M2‐like’) phenotypes existing within tumors. RT, particularly in small fractions (2 Gy or less), can promote TAMs to exert antitumor properties; for example, a study using both murine models (RT5 and human melanoma xenografts) and samples from pancreatic cancer patients receiving RT demonstrated that low‐dose RT can reprogram the differentiation of antitumor iNOS^+^ TAMs. In this study, expression of iNOS on TAMs following RT was essential to tumor vascular normalisation, T‐cell migration and tumor eradication.^52^ Similarly, a short course of low‐dose RT in human rectal cancer shifted TAMs towards enhanced phagocytic activity and expression of markers important to T‐cell activation.^53^ Results from studies exploiting the role of SIRPα on macrophages also suggest doses of 8 Gy can improve antigen presentation functions of TAMs by inducing release of immunogenic DAMPs.^54^, ^55^ To eliminate MDSCs, higher, ablative doses may be more effective, highlighting the complexities of dose selection for IO‐RT; in lung cancer and melanoma models, ablative but not conventionally fractionated RT (CFRT) decreased MDSC levels and improved tumor control and mouse survival.^56^

## The impact of radiation‐induced immunosuppressive effects

**Figure 3:**
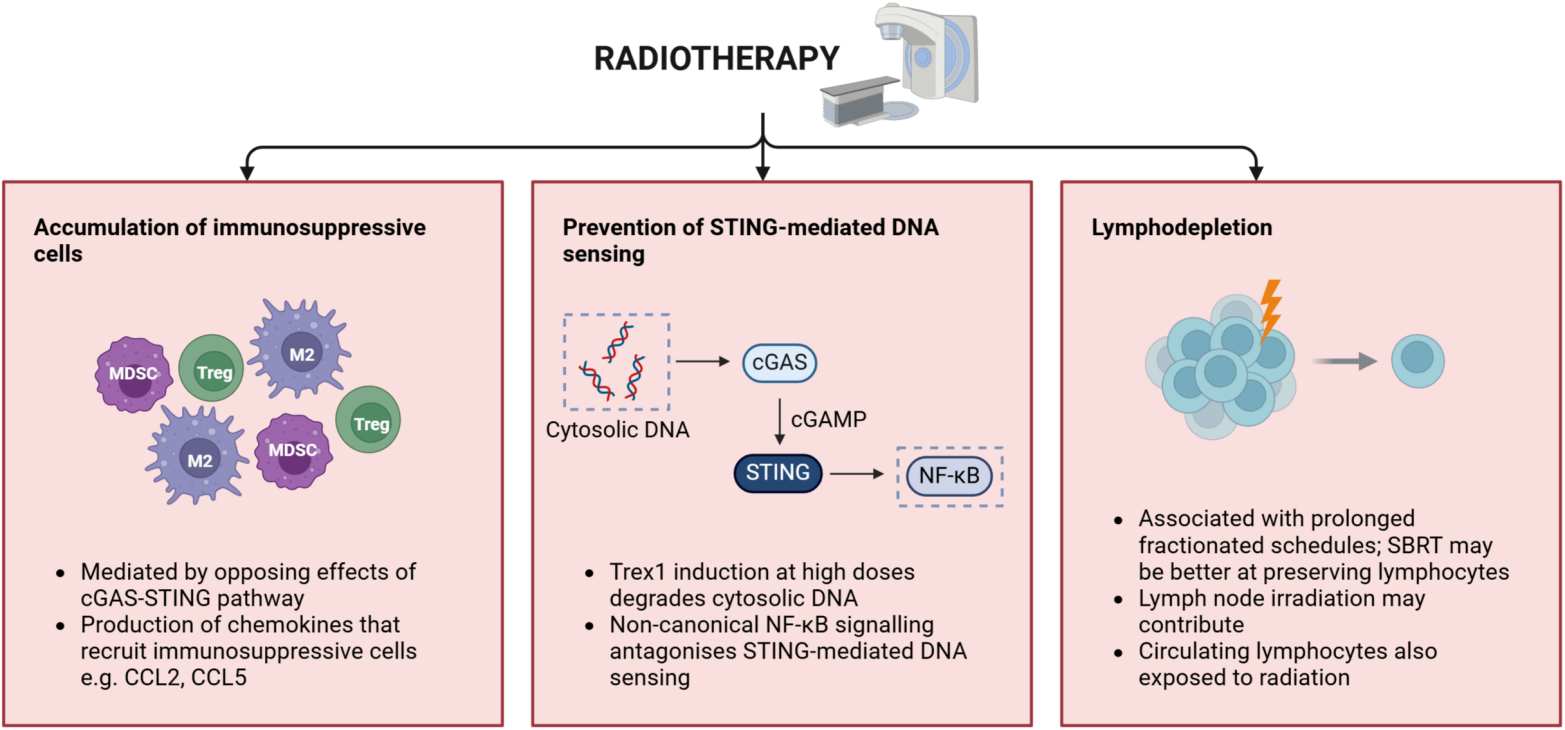
Summary of immunosuppressive effects of radiotherapy. This figure was created with Biorender.com.

Despite evidence that RT improves T‐cell activation, localisation and function, RT alone is insufficient to generate lasting antitumor immunity as it also activates immunosuppressive effects (Figure 3). These effects are at least partially dependent on RT dose; for example, in CT26 tumors, CFRT (2 Gy × 18) induced a myeloid response characterised by high numbers of MDSCs and M2‐like TAMs, while hypofractionation (8 Gy × 3) led to inflammation and a lymphoid response.^57^ In irradiated AT3‐OVA tumors, regulatory T‐cell (Treg) accumulation increased to a greater extent with high single doses (12 Gy, 20 Gy), while lower dose per‐fraction schedules (4 Gy × 3, 4 Gy × 9, 8 Gy × 3) were more effective at promoting DCs and subsequently CD8^+^ T‐cell function, which improved responses to anti‐PD1.^58^ However, immunosuppressive effects may well be inevitable regardless of dose since they appear to co‐occur with immunostimulatory effects of RT, as described further below.

First, RT‐induced activation of the cGAS‐STING pathway can potentiate opposing immune effects. For example, in oesophageal squamous cell carcinoma (ESCC) cells irradiated with 8 Gy × 3, induction of cGAS‐STING promoted CD8^+^ T‐cell infiltration, IFN‐I and expression of T‐cell chemoattractants (CXCL10, CCL5).^59^ However, cGAS‐STING was also involved in infiltration and M2 polarisation of CD163^+^ TAMs and expression of IL‐34—a cytokine promoting survival and differentiation of monocytes and macrophages. Accordingly, CD163^+^ M2 TAMs were significantly associated with IL‐34 expression in irradiated human ESCC tissues.^59^ RT‐induced increases in CCR2^+^ MDSC numbers were also found to be mediated by STING/IFN‐I activity.^60^

Additionally, while RT‐induced activation of canonical NF‐κB signalling is crucial for DC recruitment and maturation, RT also induces activation of the non‐canonical NF‐κB pathway, which suppresses STING‐mediated sensing of cytosolic DNA and subsequently inhibits the IFN‐I induction required for antitumor immune functions of DCs.^61^ Interestingly, therapeutic inhibition of non‐canonical NF‐κB signalling combined with RT (20 Gy) improved tumor growth delay compared to RT alone, which was dependent on the STING‐IFN‐I axis. Anti‐PD‐L1 further improved tumor growth delay when combined with NF‐κB inhibition and RT.^61^

While production of chemoattractants (whether by tumor or other cells in the TME) induced by RT can facilitate T‐cell infiltration, they can also recruit immunosuppressive myeloid populations. Four days after treating MC38 tumors with 15 Gy, intratumoral inflammatory Ly6C^+^CCR2^+^ monocyte numbers and proportions increased, preceded by an increase in CCL2 and CCL5 transcript and protein levels in tumors, which are ligands for the chemokine receptors CCR2 and CCR5 expressed by monocytes.^62^ CCL2 and CCL5 transcripts also increased in numerous other murine and human cancer cell lines when irradiated with 8–15 Gy *in vitro*.^28^, ^62^ Dual inhibition of CCR2/CCR5 combined with RT in MC38 tumors improved response, and this was dependent on CD8^+^ T‐cell activity.^62^

CCL2 production by tumor cells also increased in pancreatic ductal adenocarcinoma tumors following single fraction RT (14–20 Gy), leading to the recruitment of Ly6C^+^CCR2^+^ monocytes, in line with the minimal impact RT had on tumor control.^63^ Therapeutic inhibition or genetic deletion of CCL2 inhibited monocyte recruitment and improved RT efficacy. Here, RT appeared to influence the quantity, rather than the phenotype of tumor‐infiltrating monocytes, as the gene expression profile of flow‐sorted monocytes from RT‐treated tumors was minimally different from controls 1 day after RT, with only 8 of 34 365 transcripts reaching the pre‐determined threshold of two‐fold change in expression.^63^ Assessing a single time point soon after treatment may have impacted the ability to detect any effect; however, other studies have reported phenotypic changes in myeloid populations resulting from RT.

Suppressive RT effects involving lymphoid cells also impede response. For example, infiltration of TIM3‐expressing Tregs following RT and anti‐PD‐L1 was associated with resistance to treatment in orthotopic HNSCC tumors.^64^ Also, 7 days after 10 Gy, Treg proportions and absolute numbers were elevated in B16/F10, MC38 and RENCA subcutaneous tumors, and in B16/F10 this was primarily because of RT‐induced enhancement of Treg proliferation within tumors.^65^ Further phenotypic analysis revealed that expression of 4‐1BB, CTLA‐4, and the transcription factor Helios were all upregulated after RT in tumors.^65^

Lymphocytes are particularly sensitive to RT, and radiation‐induced lymphopenia (RIL) is commonly observed in cancer patients undergoing RT. Severe RIL correlates with poorer overall survival, as well as increased locoregional recurrence and metastases.^66^ LN irradiation, a common practice in cancer treatment, may exacerbate RIL and subsequently dampen the antitumor immune response. Another potential mechanism is incidental exposure of circulating lymphocytes to RT, which have higher radiosensitivity than those residing in tumor.^67^ It has been shown in CD8^+^ and CD4^+^ T cells that a dose of approximately 3 Gy reduces the surviving fraction of cells to 10% in a clonogenic survival assay.^68^ RT exposure to lymphocytes via LNs and blood is an important clinical consideration for designing effective IO‐RT strategies, discussed further in this review. In summary, taking into consideration the potential of RT‐induced immunosuppressive effects to limit the efficacy of IO‐RT is essential to optimising this therapeutic combination.

## Clinical progress combining radiotherapy with PD1, PD‐L1 and CTLA‐4 immune checkpoint inhibitors

Clinical trials of RT combined with anti‐PD1, anti‐PD‐L1 and anti‐CTLA‐4 have shown conflicting results. In the comparison of positive and negative clinical trial results below, we aim to highlight some factors, such as different clinical settings or different RT dosing, which provide at least a partial explanation for the differing results observed and provide opportunities for further enquiry.

### Conventionally fractionated radiotherapy

Immune checkpoint inhibitors can prolong responses to RT or chemoradiotherapy (CRT) in some settings. The randomised, phase III PACIFIC trial has shown that outcomes for patients with stage III NSCLC treated with definitive CRT, followed by consolidation durvalumab 1–42 days later, are markedly improved compared to patients not receiving immunotherapy.^69^ The 5‐year survival rates for those receiving durvalumab vs. placebo were estimated to be 42.9% vs. 33.4%.^69^ In a phase II single‐arm study, ESCC patients with oligometastases after first‐line ICI, and chemotherapy failure were treated with a concurrent combination of RT (40 Gy in 20 fractions for progressing or recurring primary tumors; 30 Gy in 10 fractions for new or regrowing metastases), camrelizumab (anti‐PD1) and irinotecan.^70^ A better than expected PFS and OS were observed (6.9 and 12.8 months, respectively). One of the independent prognostic factors for overall survival was the number of organs with oligometastases, suggesting IO‐RT combinations are most effective in cases of lower disease burden. The study also measured the abscopal response rate and abscopal control rate of unirradiated lesions to be 34.7% and 69.4%, respectively; this may reflect systemic immune effects of RT, or simply the activity of the systemic chemotherapy and immunotherapy in treatment. Finally, the SU2C‐SARC032 study, in patients with stage III soft tissue sarcoma of the extremity, found that the addition of preoperative and postoperative pembrolizumab to RT (50 Gy in 25 fractions) and surgery improved 2‐year disease‐free survival (52% vs. 67% in control vs. experimental groups).^71^ Larger trials will be required to confirm any benefit to overall survival and other endpoints.

Amidst these encouraging results, there have also been some disappointing trials. The PACIFIC‐2 phase III trial in unresectable stage III NSCLC assessed concurrent administration of CRT and durvalumab as a potential option for patients who discontinue CRT during treatment and are therefore not eligible for the PACIFIC regimen; however, this trial did not show a survival benefit (NCT03519971). Additionally, a phase III glioblastoma study comparing temozolomide with nivolumab for combination with RT found that nivolumab did not result in a greater survival benefit than temozolomide.^72^

### Hypofractionated radiotherapy

There is emerging evidence supporting the use of hypofractionated, ablative SBRT with PD1 ICI in early‐stage (IA‐IIB) NSCLC; Chang *et al*. conducted a randomised phase II trial for early‐stage, treatment‐naive or lung parenchymal recurrent node‐negative NSCLC, combining ablative SBRT with nivolumab. Nivolumab was commenced concurrently or within 36 h of the first RT dose, differing from the sequential scheduling used in PACIFIC.^73^ The 4‐year event‐free survival was significantly extended from 53% with RT alone to 77% with the combination, and there are several ongoing phase III trials in this setting to confirm these findings (Table 1). In the case of stage IV NSCLC where patients have acquired resistance to PD‐L1 ICI, a pooled analysis of two phase II trials suggests that hypofractionated RT may allow for temporary continuation of immunotherapy.^74^ While most patients included in these analyses received 8 Gy × 3, other dosing was used in some patients depending on the trial (ranging from 2.5 Gy × 14 to 18.75 Gy × 3), so there remains a need to confirm the ideal schedules for overcoming immunotherapy resistance.^73^, ^74^

**Table 1:** Selected ongoing clinical trials of radiotherapy and immune checkpoint inhibitors

Trial number	Phase	Cancer	Treatment	ICI target	RT administration	Primary outcome measure
NCT05765825	II (single arm)	ES‐SCLC	LDRT, concurrent cisplatin/carboplatin plus etoposide with serplulimab	PD1	Once daily fractions, 3 Gy per fraction, to a target dose of 15 Gy in 5 fractions	PFS
NCT04214262	III	NSCLC, stage I–IIA, unresectable	Atezolizumab + SBRT vs. SBRT alone	PD‐L1	Every 2 days, SBRT for 3–8 treatments (dose not specified), beginning day 1 of cycle 3 of atezolizumab	OS
NCT03833154 (PACIFIC‐4)	III	NSCLC, stage I–II, unresected	Durvalumab + SBRT vs. SBRT alone; also osimertinib following SBRT in patients with EGFR mutation	PD‐L1	Definitive SBRT prior to durvalumab	PFS (main cohort); 4‐year PFS (EGFR cohort)
NCT04402788 (RAPTOR)	II–III	ES‐SCLC	Atezolizumab + nonablative consolidative radiation vs. atezolizumab alone, after completion of atezolizumab plus chemotherapy	PD‐L1	RT once daily on days 1–5 during weeks 1–5	PFS (phase II), OS (phase III)
NCT05484024 (STELLAR II)	II–III	Locally advanced rectal cancer	Short‐course RT followed by neoadjuvant chemotherapy and sintilimab	PD1	5 Gy per fraction, to a target dose of 25 Gy in 5 fractions, followed by systemic treatments 14 days later	Complete remission, disease‐free survival rate
NCT05024318 (NAPSTER)	II	Renal cell carcinoma	Pembrolizumab followed by SBRT after cycle 1 + nephrectomy vs. SBRT + nephrectomy alone	PD1	42 Gy in 3 fractions, completed within 2–3 weeks	Major pathological response, changes in CD8^+^ T_RM_ and/or TCF1^+^ T cells from pre‐treatment biopsy to post‐nephrectomy
NCT03961971	I	Recurrent glioblastoma multiforme	Spartalizumab and MGB453 followed by stereotactic radiosurgery	PD1, TIM3	Stereotactic radiosurgery	Number of participants with serios adverse events, as defined by NCI CTC v5.0
NCT05743504	Ib/II	Locally advanced oesophageal squamous cell carcinoma	Neoadjuvant tiragolumab, atezolizumab, paclitaxel, cisplatin and radiotherapy followed by surgery	TIGIT, PD1	CFRT, dose unspecified	Pathological complete response rate
NCT03610711 (REACTION)	Ib	Recurrent or metastatic gastroesophageal cancer	SBRT to multiple metastatic sites plus nivolumab with or without relatlimab	PD1, LAG3	24 Gy in 3 fractions, followed by immunotherapy	Change in the infiltrating CD8^+^ T cell density units after systemic treatment with radiation plus nivolumab with or without relatlimib
NCT03044613	Ib	Operable stage II–III oesophageal/gastroesophageal junction cancer	2 cycles of induction nivolumab prior to concurrent chemoradiation plus nivolumab or nivolumab + relatlimab before surgery	PD1, LAG3	CFRT, dose unspecified	Number of participants with treatment‐related adverse events as assessed by CTCAE v4.0

In contrast, CHEERS, a phase II randomised trial in advanced solid cancers, failed to show a survival benefit of adding subablative, hypofractionated SBRT to a limited number of tumor lesions (8 Gy × 3) with PD1 or PD‐L1 ICI. A potential caveat to this study is that a large proportion of patients (68%) also had previous exposure to systemic treatment.^75^ In another randomised, phase II trial in Merkel cell carcinoma assessing dual ICI (nivolumab and ipililumab) with or without SBRT (8 Gy × 3), the addition of SBRT did not further improve ORR in those with previous ICI exposure.^76^ An early phase trial in unresectable, locally advanced pancreatic cancer combining SBRT (8 Gy × 4) with nivolumab also did not achieve long‐term treatment responses.^77^

### Low‐dose radiotherapy

The use of LDRT to prime the TME for favorable ICI outcomes has a clear advantage over other, more aggressive regimens from the perspective of preventing toxicity, as well as clinical feasibility. Work by Wang *et al*. supports a role for low‐dose, short‐course RT (LDRT) in extensive‐stage small‐cell lung cancer (ES‐SCLC).^78^ The phase II MATCH trial assessed the combination of LDRT (3 Gy × 5) with atezolizumab (anti‐PD‐L1) and chemotherapy, in the first‐line setting for ES‐SCLC. This treatment schedule exhibited promising antitumor activity and potential survival benefit, with an objective response rate (ORR) of 87.5% and median PFS of 6.9 months. In a corresponding preclinical study using two separate models of ES‐SCLC, one key mechanism of LDRT was to recruit stem‐like (TCF1^+^PD1^+^) CD8^+^ T cells from lymph nodes into the tumor. In line with this, single‐cell RNA sequencing data from biopsy samples (two from patients receiving LDRT + chemoimmunotherapy and two only receiving chemoimmunotherapy) showed the proportion of stem‐like CD8^+^ T cells was increased in LDRT‐treated tumors. Overall, CD8^+^ T cells also showed higher stemness and cytotoxicity scores, and lower exhaustion scores with LDRT.^78^ A similar trial is validating the use of anti‐PD1 instead of anti‐PD‐L1 in this setting (Table 1). The highly proliferative, and thus more radiosensitive, nature of ES‐SCLC may mean that LDRT could have greater benefit in this cancer than others, since in a phase II trial of metastatic NSCLC refractory to PD1 or PD‐L1 ICI, the addition of LDRT did not further improve the response to dual ICI with durvalumab and tremelilumab.^79^ However, an early phase trial in treatment‐naïve, PD‐L1^+^ stage IV NSCLC showed acceptable safety and promising activity of combining ablative SBRT with LDRT and sintilimab (anti‐PD1),^80^ the rationale being that combining high and low doses could achieve both tumor shrinkage and TME priming. Nevertheless, more clinical data on combining LDRT with ICI is required.

## Considerations for optimising IO‐RT combinations

While there have been some advances made with IO‐RT in the clinical setting, positive results described above are modest at best, and numerous trials have failed to show therapeutic benefit. In part, this may be explained by differences in RT dosing; for example, several negative trials discussed above used sub‐ablative regimens in advanced malignancy, and the immunostimulatory effects of RT may have been insufficient to effect robust tumor regression in this setting. At the same time, tissue‐agnostic cancer trials, such as CHEERS, may impede the ability to detect any treatment effect because of heterogeneity across cancer types. Furthermore, in successful trials utilising CFRT and ICI, adjunct chemotherapy or surgery also forms part of the treatment plan—making it difficult to distinguish the relative contribution of RT immune effects. Clinical evidence also suggests hypofractionation is perhaps less prone to exerting immunosuppressive effects such as lymphodepletion than CFRT. Overall, continued research into the effects of RT dosing and fractionation will be essential to optimising IO‐RT combinations; however, there are also other aspects beyond this to be considered.

Despite being common practice in cancer treatment to prevent recurrence or spread, LN irradiation can impede RT‐induced immune responses, as demonstrated in muscle‐invasive bladder cancer and HNSCC.^81^, ^82^ However, other studies have found that completely omitting LN irradiation leads to unacceptably high recurrence, demonstrating the importance of this practice for addressing potential outgrowth from subclinical disease originating from LNs.^83^ While the detrimental effect of LN irradiation is often attributed to the development of RIL, it has also been proposed that LN irradiation disrupts the CCR7‐CCL19/CCL21 immune cell homing axis, leading to poor DC homing to LNs and thus reduced T‐cell activation.^84^ In this study, Telarovic *et al*. found that delayed LN irradiation led to superior tumor control over concomitant or neoadjuvant LN irradiation in mouse tumors receiving RT + ICI.^84^ Additional work is required to determine when LN‐sparing approaches would be beneficial in the context of IO‐RT.

The role of RT exposure to circulating lymphocytes in causing RIL and its potential impact on treatment efficacy should also be considered. A model developed by Yovino *et al*. to estimate the blood dose from radiation exposure suggested that the overall proportion of circulating lymphocytes exposed to RT correlates with target volumes, field size and fractionation regimen.^85^ Consistent with this, Wild *et al*. demonstrated the lymphocyte‐sparing effects of hypofractionated RT (6.6 Gy × 5) over CFRT (1.8 Gy × 28) in patients with locally advanced pancreatic cancer.^86^ Additionally, in an analysis of lung cancer patients, 3 Gy × 15 but not 12.5 Gy × 4 was predictive of a decline in absolute lymphocyte count, which in turn predicted survival of patients receiving CFRT.^87^ These data collectively support the use of hypofractionation to preserve lymphocytes.

Incorporating biomarker‐based selection of patients who will benefit the most from IO‐RT will be crucial to optimising IO‐RT combinations. Peripheral blood biomarkers are becoming increasingly relevant owing to the ease of dynamic sample collection, as well as innovative sequencing technologies increasing the rate and throughput of candidate biomarker discovery. A preliminary study in the context of ablative SBRT + ICI for oligoprogressive metastatic cancer has identified potential biomarkers in circulating cell‐free DNA (cfDNA) and small RNA from extracellular vesicles.^88^ Here, peripheral blood samples were collected at baseline (T1), after the first (T2) and last (T3) RT fraction, with decreased cfDNA from T2 to T3 correlating with a good response. Furthermore, at T2, CD8^+^PD‐L1^+^ and CD8^+^PD1^+^ cells were increased in responders and non‐responders, respectively, with 27 microRNAs also differentially expressed.^88^ A study in melanoma also identified a baseline T‐cell signature of lower levels of naïve CD8^+^ T cells and higher TIM3 expression on Tregs and memory T cells, which predicted response to ablative SBRT and ICI.^89^

Finally, comprehensive testing of ideal IO‐RT schedules will not be achieved through *in vivo* research alone. To address this, there has been increasing interest in *in silico* exploration of immune interactions and the efficacy of IO‐RT.^90^ Being able to model different cancer and patient settings, informed by the existing body of *in vivo* data, can help consolidate findings from studies in IO‐RT, generate hypotheses and accelerate translation.

## Emerging IO‐RT combinations

Each of the considerations discussed above suggests that IO‐RT will require a personalised approach, dependent on the clinical setting and likelihood of response. The development of alternative ICIs and dual ICI combinations is one strategy to maximise patient benefit, and early‐phase clinical trials of RT combined with emerging ICIs, including TIM3, TIGIT and LAG3, are currently ongoing (Table 1). Preclinical studies have also demonstrated the potential of combining RT and agonistic antibodies targeting stimulatory immune checkpoints such as GITR, OX40, 41‐BB and CD40.^2^, ^91^, ^92^, ^93^ Moreover, novel approaches such as cancer vaccines and CAR T cell therapy represent critical advances in the era of immunotherapy, and evidence suggests they may be further enhanced by RT, expanding the range of viable treatment options for patients.

### Cancer vaccines and radiotherapy

Personalised cancer vaccines are an emerging strategy to elicit *de novo* T‐cell activation against tumor‐specific or tumor‐associated antigens. Lhuillier *et al*. provide proof‐of‐concept evidence for a role of RT in improved personalised neoantigen vaccination strategies, but upregulating the expression of mutated genes.^30^ In 4T1 cells, RT (8 Gy × 3) upregulated three genes (*Dhx58, Cand1* and *Adgrf5*) encoding two MHC‐I and one MHC‐II immunogenic neoepitopes. Vaccination with these neoepitopes elicited neoantigen‐specific CD8^+^ and CD4^+^ T‐cell responses, which were both required for vaccine efficacy.^30^ The ongoing phase II iTAPVR study (NCT06314087) is assessing an individualised tumor neoantigen peptide vaccine with RT for patients with advanced solid tumors.

Vaccine strategies involving induced pluripotent stem cells (iPSCs) have gained considerable attention, as iPSCs share tumor‐associated antigen profiles with various cancers.^94^ However, while they have demonstrated the ability to induce protective, tumor‐specific responses prophylactically, they are insufficient in a therapeutic setting. However, a recent study has shown that combining neoantigen‐augmented, iPSC‐based (NA‐iPSC) vaccines with RT (5 Gy × 3) enhances tumor control and inhibits distant metastases in CT26 and 4T1 cancer models, through increasing neoantigen‐specific CD8^+^ T‐cell responses, IFNγ and granzyme B production, and STING‐mediated infiltration of DCs, CD8^+^ T cells, and NK cells.^95^ Overall, current evidence suggests that neoantigen vaccination could play a role in enhancing responses to RT and ICI.

### CAR‐T cell therapy and radiotherapy

The use of RT is a promising strategy to overcome mechanisms of CAR‐T cell failure in solid tumor, including poor localisation and homing; poor expression of tumor neoantigen; poorly functioning tumor vasculature; and hypoxia‐induced dysfunction. Current evidence seems to suggest that the ideal RT schedules for combination with CAR‐T cells are subablative, to exert specific immune‐activating effects rather than reducing the tumor load. For example, in a model of pancreatic adenocarcinoma heterogeneously expressing sialyl Lewis‐A (sLeA), 2 Gy led to increased susceptibility of both sLeA^+^ and sLeA^−^ tumor cells to sLeA‐CAR‐T cell killing, mediated by RT‐induced sensitivity to TRAIL produced by sLeA‐CAR‐T cells.^96^ RT‐induced CAR‐T cell tumor infiltration has also been observed to lead to superior tumor control; in orthotopic murine models of glioblastoma (GL26 and SB28 cell lines engineered to express GD2), treatment with 5 Gy radiation and GD2 CAR‐T cells led to complete responses not seen with either treatment alone.^97^ Imaging of these tumors in real time revealed that RT led to rapid extravasation of CAR‐T cells from the vasculature and increased expansion. Interestingly, concurrent but not sequential therapy resulted in these effects.^97^ In an orthotopic mesothelioma model, pretreatment with 4 Gy thoracic RT enhanced chemotaxis, tumor infiltration, proliferation, memory and persistence of mesothelin‐targeted CAR‐T cells.^98^ Further supporting a role of subablative RT to improve CAR‐T cell efficacy, studies in a bilateral pancreatic (Panc02) tumor model showed that the combination of 4 Gy RT to one tumor plus CLDN18.2‐specific CAR‐T therapy led to abscopal effects.^99^ More research is required to answer questions such as whether CAR‐T cells exhibit similar sensitivity to RT as CD8^+^ or CD4^+^ T cells, whether RT may be used to restore CAR‐T phenotype following relapse, or the ideal timing of RT relative to CAR‐T cells, to avoid potentially irradiating CAR‐T cells in the treatment field. Currently, clinical trials combining CAR‐T cells and RT are being conducted mostly in large B‐cell lymphomas or multiple myeloma (NCT06104592, NCT05621096, NCT05514327 and NCT06623630), with one trial assessing mesothelin‐targeted CAR‐T therapy in patients with mesothelioma (NCT04577326), the results of which are eagerly awaited.

## Conclusion

Preclinical study has demonstrated the coexistence of immunostimulatory and immunosuppressive effects of RT, which may play a role in the mixed findings of IO‐RT clinical trials to date.^100^ Although the translation of IO‐RT into patient care has proved to be a formidable challenge, clinical data to date have provided valuable lessons. Developing accurate biomarkers and addressing clinical considerations raised in this review will help refine and personalise the approach to IO‐RT, maximising success. Mechanistic preclinical studies have formed a strong foundation for the growth of the IO‐RT field, and preclinical study will continue to be necessary to understand immune dynamics underlying clinical findings, identify candidate biomarkers, and optimise combination of RT with other immunotherapies such as cancer vaccines and CAR‐T therapy. Overall, as RT improves in safety and precision, and more immunotherapeutic agents continue to emerge, IO‐RT is sure to provide additional treatment options to benefit more cancer patients.

## Author contributions

**Faith Hartley:** Conceptualization; writing – original draft; writing – review and editing. **Martin Ebert:** Supervision; writing – review and editing. **Alistair M Cook:** Supervision; writing – review and editing.

## Conflict of interest

The authors declare no conflict of interest.
